# An Interval Iteration Based Multilevel Thresholding Algorithm for Brain MR Image Segmentation

**DOI:** 10.3390/e23111429

**Published:** 2021-10-29

**Authors:** Yuncong Feng, Wanru Liu, Xiaoli Zhang, Zhicheng Liu, Yunfei Liu, Guishen Wang

**Affiliations:** 1 College of Computer Science and Engineering, Changchun University of Technology, Changchun 130012, China; fengyuncong@ccut.edu.cn (Y.F.); lwrtime@163.com (W.L.); liuzhicheng22020@163.com (Z.L.); lyf199612113@163.com (Y.L.); wangguishen@ccut.edu.cn (G.W.); 2 Artificial Intelligence Research Institute, Changchun University of Technology, Changchun 130012, China; 3 Key Laboratory of Symbolic Computation and Knowledge Engineering of Ministry of Education, Jilin University, Changchun 130012, China; 4 College of Computer Science and Technology, Jilin University, Changchun 130012, China

**Keywords:** image segmentation, multilevel thresholding, interval iteration, layer decomposition, segmentation fusion

## Abstract

In this paper, we propose an interval iteration multilevel thresholding method (IIMT). This approach is based on the Otsu method but iteratively searches for sub-regions of the image to achieve segmentation, rather than processing the full image as a whole region. Then, a novel multilevel thresholding framework based on IIMT for brain MR image segmentation is proposed. In this framework, the original image is first decomposed using a hybrid *L*_1_ − *L*_0_ layer decomposition method to obtain the base layer. Second, we use IIMT to segment both the original image and its base layer. Finally, the two segmentation results are integrated by a fusion scheme to obtain a more refined and accurate segmentation result. Experimental results showed that our proposed algorithm is effective, and outperforms the standard Otsu-based and other optimization-based segmentation methods.

## 1. Introduction

Image segmentation is a key step in image processing and image analysis [1,2,3]. The process of image segmentation refers to dividing an image into several disjoint regions based on features such as intensity, color, spatial texture, and geometric shapes, so that these features show consistency or meaningful similarity in the same region, but show obvious differences between different regions [4,5]. Image segmentation is widely used in many fields, such as computer vision, object recognition, and medical image applications [6,7].

In the field of medical research and practice, image segmentation technology can be applied to computer-aided diagnosis, clinical surgical image navigation, and image-guided tumor radiotherapy [8,9]. Segmentation of organs and their substructures from medical images can be used to quantitatively analyze clinical parameters that are related to volume and shape [10]. For instance, a brain MR image can be segmented into five main regions, namely, the gray matter (GM), white matter (WM), cerebrospinal fluid (CSF), the skull, and the background. In diagnosis of brain disease, WM abnormalities are closely related to multiple sclerosis, schizophrenia, and Alzheimer’s disease. Autism is relevant to changes in the volume of the GM [8,11]. Central nervous system lesions and metabolic disorders of nerve cells change the properties and composition of CSF. When the central nervous system is damaged, the detection of CSF is one of the important auxiliary diagnostic methods. Therefore, accurate segmentation of different object regions in a brain MR image is believed to be one of the most significant tasks for clinical research and treatment.

A large number of image segmentation methods have been previously researched. In [12], Fu et al. classified image segmentation techniques, such as characteristic feature thresholding [13,14,15] or clustering [16,17], edge detection [18,19], and region extraction [20,21]. Other approaches include graph cut methods [22,23] and deep neural network-based methods [24]. Among the existing segmentation methods, thresholding is considered to be an efficient and popular techniques because of its simplicity and high efficiency [25,26,27,28]. Thresholding can be classified into two groups: bi-level thresholding and multi-level thresholding [29]. The former segments an original image into two regions (foreground and background) by searching for an optimal threshold based on gray histogram. Pixels with gray values greater than the threshold are classified as the foreground, whereas pixels with gray values lower than the threshold are classified as the background. When such a simple binary classification is insufficient for subsequent processing, bi-level thresholding is extended to multi-level thresholding, which refers to partitioning the image into several different regions using more thresholds [30].

He et al. proposed an efficient krill herd method to identify optimal thresholding values by maximizing three different objective functions: between-class variance, Kapur’s entropy, and Tsallis entropy [29]. Lei et al. defined square rough entropy in a new form, and presented a novel image segmentation thresholding method based on minimum square rough entropy [31]. The optimal threshold was selected as the value that made the roughness of the object region and the background zero. Yan et al. proposed a novel multilevel thresholding using Kapur’s entropy based on the whale optimization algorithm [32]. This can overcome premature convergence and obtain the global optimal solution. Singh proposed an adaptive thresholding algorithm based on neutrosophic set theory for segmenting Parkinson’s disease MR images [33]. The gray value that maximizes neutrosophic entropy information is selected as the optimal threshold. Omid Tarkhaneh et al. presented a differential evolution-based multilevel thresholding algorithm for MR brain image segmentation [34]. Inspired by Levy distribution, Cauchy distribution, and Cotes’ Spiral, a novel mutation scheme was designed to model swarm intelligence optimization. To solve the increasing complexity of optimization problems, Zhao et al. proposed an improved ant colony optimization algorithm based on the chaotic random spare strategy for multilevel thresholding [35]. The random spare strategy was applied to improve the convergence speed, and the chaotic intensification strategy was used to improve the convergence accuracy and avoid falling into a local optimum. Cai et al. proposed an iterative triclass Otsu thresholding algorithm for microscopic image segmentation [36]. In contrast to the standard Otsu method, it firstly segments an original image into the foreground, the background, and a third region, namely, the “to-be-determined (TBD)” area, based on two class means as obtained by Otsu’s optimal threshold. Then, similar processing is iteratively applied to the TBD region until the preset criterion is met. This single thresholding method performs well for weak objects and segmentation of fine details, but is not applicable to complicated medical image segmentation. However, medical image segmentation is still regarded as an important yet challenging work due to the complexity of the medical image itself, such as low tissue contrast, irregular shape, and large location variance [37].

To improve the quality of image segmentation, we proposed an interval iteration-based multilevel thresholding algorithm for brain MR images. In the algorithm, hybrid *L*_1_ − *L*_0_ layer decomposition is adopted to reduce the influence of noise on the segmentation effect. Traditional Otsu multilevel thresholding processes the full image as a whole region, and is inclined to the class with a large variance. To overcome this problem, we extended Cai’s method [36] to multilevel thresholding and proposed a novel interval iteration method to identify optimal thresholds. In addition, a fusion strategy is used to integrate different segmentation images to obtain finer segmentation results. In general, the key contributions of our work can be summarized as follows:

(1) A hybrid *L*_1_ − *L*_0_ layer decomposition method is used to achieve the base layer of an original image, which can remove noise and preserve edge information in the segmentation process.
(2) An interval iteration multilevel thresholding method is proposed in this paper. In the grayscale histogram of an original image, iterations are separated by the combination of class means and thresholds, and Otsu single thresholding is iteratively applied to each iteration.
(3) A fusion strategy is adopted to fuse different segmentation results. It takes both spatial and intensity information into account, and makes segmentation more accurate.

The rest of this paper is organized as follows. Section 2 details the interval iteration-based multilevel thresholding method. The framework of the proposed algorithm and related processing are described in Section 3. Section 4 depicts the experiments on brain MR image segmentation including results and analysis. Finally, conclusions and future work are presented and discussed in Section 5.

## 2. Interval Iteration Based Multilevel Thresholding

In this section, we propose a novel multilevel thresholding algorithm based on interval iteration. The iterative process is illustrated in the following.

### 2.1. Otsu Method

Let *I* be an image with size of *M* × *N*, and the gray level denoted as $G=\left\{0,\;1,\;\ldots ,\;255\right\}$. We define *n_j_* as the number of pixels with gray level *j*, and define $P_{j}=\frac{n_{j}}{M\times N}\;,\;(p_{j}\geq 0,\;j\in G)$ as the probability of such pixels, in which $\sum_{j=0}^{255}P_{j}=1$. Assuming that *I* is to be segmented into *K* + 1 (K≥1) classes ($C_{1},\;C_{2},\;\ldots ,\;C_{K+1}$) by *K* thresholds ($t_{1},t_{2},\;\ldots ,\;t_{K}$), the Otsu method searches the histogram of *I* to find one or more thresholds that minimize intra-class variance or maximize the between-class variance, i.e., Otsu can be defined as $\{T_{1},T_{2},\;\ldots ,\;T_{K}\}=$$\underset{0\leq t_{1}<t_{2}<\ldots <<t_{K}\leq L}{arg\;max}\{\sigma_{B}^{2}(t_{1},t_{2},\;\ldots ,\;t_{K})\}$. If *K* = 1, it is referred to as single thresholding; otherwise, multilevel thresholding. The between-class variance $\sigma_{B}^{2}$ is calculated as follows:

(1) $$\sigma_{B}^{2}(t_{1},t_{2},\cdots t_{K})=\sum_{i=1}^{K+1}\omega_{i}{\left(\mu_{i}-\mu_{T}\right)}^{2}$$

where $\omega_{i}$ and $\mu_{i}$ denote the probability and mean of class *C_i_*, respectively.

(2) $$\begin{array}{l} \left\{\begin{array}{l} \omega_{1}=\sum_{j=0}^{t_{1}}P_{j}\\ \omega_{i}=\sum_{j=t_{i-1}+1}^{t_{i}}P_{j}\;,(i=2,\ldots ,K)\\ \omega_{K+1}=\sum_{j=t_{K}+1}^{255}P_{j} \end{array}\right. \end{array}$$

(3) $$\left\{\begin{array}{l} \mu_{1}=\sum_{j=0}^{t_{1}}j\cdot \frac{P_{j}}{\omega_{1}}\\ \mu_{i}=\sum_{j=t_{i-1}+1}^{t_{i}}j\cdot \frac{P_{j}}{\omega_{i}}\;,(i=2,\ldots ,K)\\ \mu_{K+1}=\sum_{j=t_{K}+1}^{255}j\cdot \frac{P_{j}}{\omega_{K+1}} \end{array}\right.$$

$\mu_{T}$ represents the total mean of *K* + 1 classes.

(4) $$\mu_{T}=\sum_{j=0}^{255}j\cdot P_{j}$$

### 2.2. Interval Iteration Based Multilevel Thresholding

#### 2.2.1. The First Iteration

**Figure 1:**
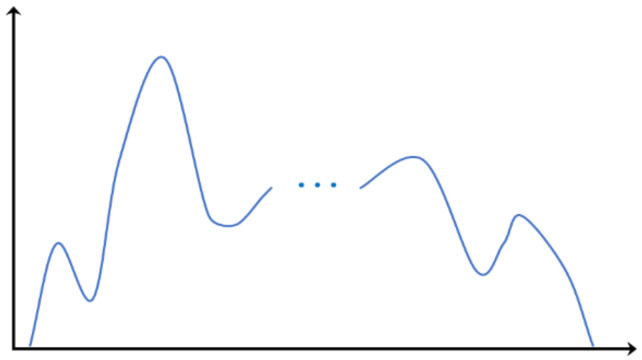
The gray histogram curve of an original image.

Given an original image *I*, we can obtain its gray histogram curve. Here, an artificial example is shown in Figure 1.

**Figure 2:**
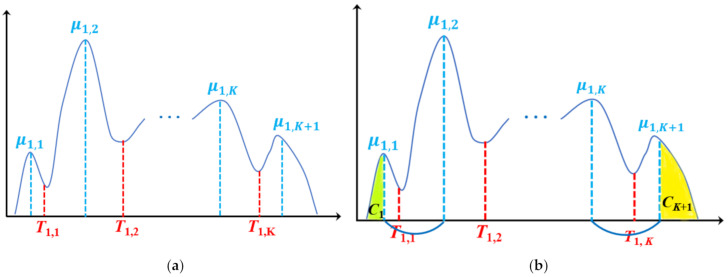
Thresholds (*T*_1,1_, *T*_1,2_, …, *T*_1,***K***_) and class means (*μ*_1,1_, *μ*_1,2_, …, *μ*_1,***K***_, *μ*_1,***K*+1**_) from the first iteration: (**a**) thresholds and class means, (**b**) two divided classes *C*_1_ and *C_K_*_+1_.

In the first iteration, traditional Otsu multilevel thresholding is performed on the original image to search for *K* thresholds. *K* + 1 class means and *K* initial thresholds can be achieved by computing Equation (1). Figure 2 illustrates the results of Otsu multilevel thresholding. In Figure 2a, *K* + 1 class means are denoted as *μ*_1,*i*_ (*i* = 1, …, *K* + 1), and *K* initial thresholds are denoted as *T*_1,*i*_, (*i* = 1, …, *K*). Then, we design a manner of classification. Pixels whose gray values satisfy $p\leq \mu_{1,1}$ are partitioned into class *C*_1_; pixels whose gray values satisfy $q\geq \mu_{1,K+1}$ are partitioned into class *C_K_*_+1_. The remaining pixels are divided into *K* intervals [*μ*_1,1_, *μ*_1,2_], [ *μ*_1,2_, *μ*_1,3_], …, [*μ*_1,*K*_, *μ*_1,*K*+1_] according to their gray values, and they are classified in the next iteration. Figure 2b shows an example of the classification. In Figure 2b, the green part denotes *C*_1_ and the yellow part represents *C_K_*_+1_; the part between *C*_1_ and *C_K_*_+1_ needs to be determined in subsequent iterations.

#### 2.2.2. The Second Iteration

**Figure 3:**
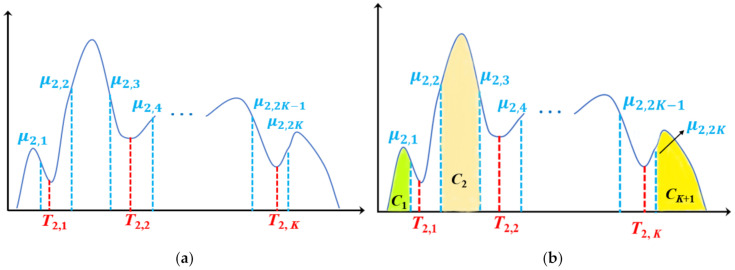
Thresholds (*T*_2,1_, *T*_2,2_, …, *T*_2,***K***_) and class means (*μ*_2,1_, *μ*_2,2_, …, *μ*_2,2***K***_) from the second iteration: (**a**) thresholds and class means, (**b**) *K*+1 divided classes *C*_1_, *C*_2_, …, *C_K_*_+1_.

In the second iteration (as shown in Figure 3), new thresholds *T*_2,*i*_ (*i* = 1, …, *K*) are obtained by applying Otsu single thresholding to *K* intervals [*μ*_1,1_, *μ*_1,2_], [ *μ*_1,2_, *μ*_1,3_], …, [*μ*_1,*K*_, *μ*_1,*K*+1_], respectively. Furthermore, two class means *μ*_2,2*i*−1_, *μ*_2,2*i*_ are obtained by *T*_2,*i*_ in [*μ*_1,*i*_, *μ*_1,*i*+1_] (*i* = 1, …, *K*), which are shown in Figure 3a. Then, classes *C*_1_ and *C_K_*_+1_ are updated by adding new pixels whose gray values are in the intervals [*μ*_1,1_, *μ*_2,1_] and [*μ*_2,2*K*_, *μ*_1,*K*+1_]. These are shown as the green and yellow parts in Figure 3b, respectively. Alternatively, pixels whose gray values are in the intervals [*μ*_2,2_, *μ*_2,3_], [ *μ*_2,4_, *μ*_2,5_], …, [*μ*_2,2*K*−2_, *μ*_2,2*K*−1_] are divided into *K* − 1 classes *C*_2_, …, *C_K_*, respectively (shown as the light orange part in Figure 3b).

#### 2.2.3. The *s*th Iteration

**Figure 4:**
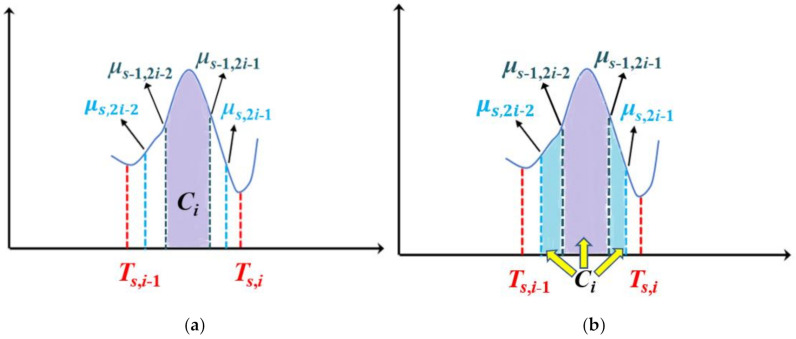
An example of updating class *C_i_*: (**a**) the divided class *C_i_* in the (*s*-1)th iteration, (**b**) the updated class *C_i_* in the *s*th iteration.

In the *s* (*s* ≥ 3) iteration, new thresholds *T_s,i_* (*i* = 1, …, *K*) are respectively obtained by applying the Otsu method to intervals [*μ_s_*_−1,1_, *μ_s_*_−1,2_], [ *μ_s_*_−1,3_, *μ_s_*_−1,4_], …, [*μ_s_*_−1,2*K*−1_, *μ_s_*_−1,2*K*_] which are produced from the previous iteration. Two class means *μ_s_*_,2*i*−1_, *μ_s_*_,2*i*_ are obtained by *T_s_*_,*i*_ in the interval [*μ_s_*_−1,2*i*−1_, *μ_s_*_−1,2*i*_] (*i* = 1, …, *K*). New pixels are added to classes *C*_1_, *C*_2_, …, *C_K_*_+1_, respectively; *C*_1_ and *C_K_*_+1_ are expanded by adding new pixels whose gray values are in the intervals [*μ_s_*_−1,1_, *μ_s_*_,1_] and [*μ_s_*_,2*K*_, *μ_s_*_−1,2*K*_], respectively. *C_i_* (*i* = 2, …, *K* − 1) is expanded by adding new pixels whose gray values are in the intervals [*μ_s_*_,2*i*−2_, *μ_s_*_−1,2*i*−2_] and [*μ_s_*_−1,2*i*−1_, *μ_s_*_,2*i*−1_]. For clarity, an example of the process to update class *C_i_* is displayed in Figure 4. In Figure 4a, *μ_s_*_−1,2*i*−2_ and *μ_s_*_−1,2*i*−1_ are two class means obtained from the (*s*−1)th iteration, in which iteration class *C_i_* includes pixels whose gray values are in the interval [*μ_s_*_−1,2*i*−2_, *μ_s_*_−1,2*i*−1_] (shown as the purple part). In Figure 4b, *μ_s_*_,2*i*−2_ and *μ_s_*_,2*i*−1_ are two new class means obtained from the *s*th iteration. Pixels in the two intervals [*μ_s_*_,2*i*−2_, *μ_s_*_−1,2*i*−2_] and [*μ_s_*_−1,2*i*−1_, *μ_s_*_,2*i*−1_] (two blue areas) are divided into class *C_i_*.

**Figure 5:**
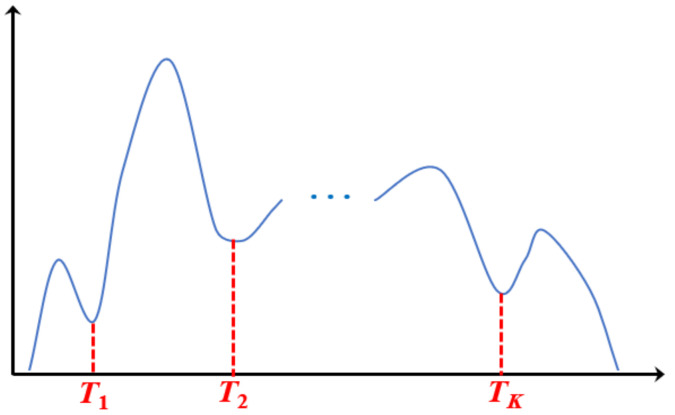
All the obtained optimal thresholds *T*_1_, *T*_2_, …, *T_K_*.

The above process is repeated, and the search for the *r*th threshold is stopped if the difference between two consecutive thresholds is less than δ (δ>0), i.e., $|T_{h,r}-T_{h-1,r}|\;<\delta$. Then the *r*th optimal threshold is set as $T_{r}=T_{h,r}$. The iteration is stopped when all the optimal thresholds *T*_1_, *T*_2_, …, *T**_K_*** (as shown in Figure 5) are found.

Algorithm 1 summarizes the framework of interval iteration-based multilevel thresholding (IIMT).


**Algorithm 1.** Interval iteration-based multilevel thresholding (IIMT).**Input:** original image *I*, number of thresholds *K* (K≥2), constant δ (δ>0);**Output:** optimal thresholds *T*_1_, *T*_2_, …, *T**_K_***;1:  Otsu multilevel thresholding (maximize Equation (1)), obtain thresholds *T*_1,1_, *T*_1,2_, …,  *T*_1,***K***_, corresponding class means *μ*_1,1_, *μ*_1,2_, …, *μ*_1,***K***_, *μ*_1,***K*+1**_, and divided classes *C*_1_, *C_K_*_+1_;2:  Otsu single thresholding in interval [*μ*_1,*i*_, *μ*_1,*i*
**+** 1_] (*i* = 1, …, *K*), obtain corresponding  threshold *T*_2,*i*_, and class means *μ*_2,2*i*−1_, *μ*_2,2*i*_, update classes *C*_1_, *C_K_*_+1_, obtain divided  classes *C*_2_, …, *C_K_*;3:  **for** *i* = 1, …, *K* **do**4:     *s* = 3;5:     **do**6:     {Otsu single thresholding in every interval [*μ_s_*_−1,2*i*−1_, *μ_s_*_−1,2*i*_] (*i* = 1, …, *K*), obtain corresponding threshold *T_s_*_,*i*_ and class means *μ_s_*_,2*i*−1_, *μ_s_*_,2*i*_, update divided classes *C_i_*;7:     *s*++;8:    } **while** ($|T_{s-1,i}-T_{s-2,i}|\;<\delta$)9:   $T_{i}=T_{s-1,i}$;10:  **end for**


## 3. The Proposed Algorithm

### 3.1. The Framework

**Figure 6:**
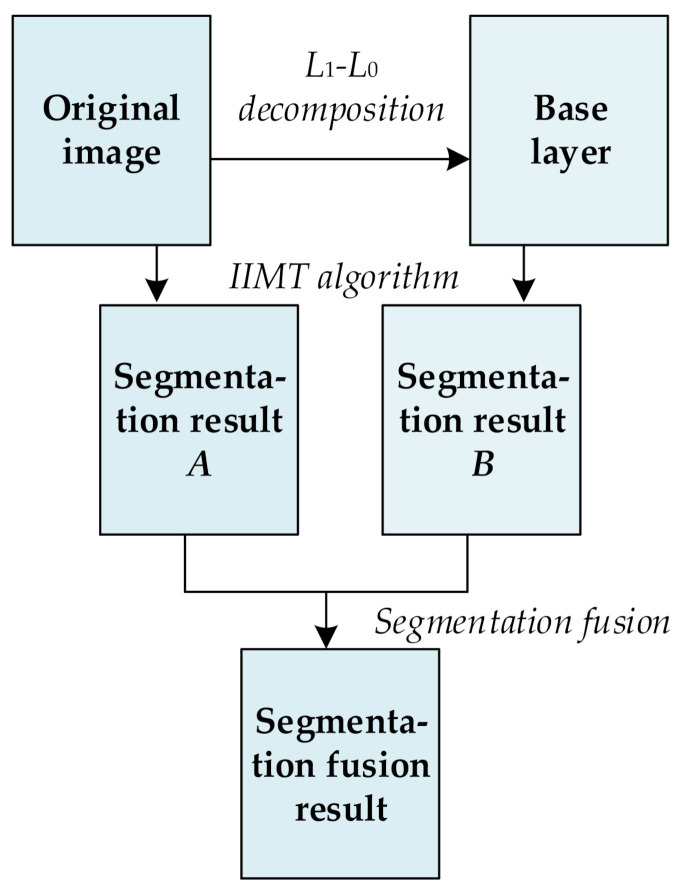
Framework of the proposed algorithm.

The framework of the proposed algorithm is shown in Figure 6. It is illustrated as follows.

(1) A hybrid *L*_1_ − *L*_0_ layer decomposition method is performed on the original image to obtain its base layer.
(2) The original image and its base layer are segmented by the IIMT algorithm, and their segmentation results are denoted *A* and *B*, respectively.
(3) The segmentation fusion method is applied to *A* and *B* to obtain the final segmentation result.

### 3.2. Hybrid L_1_ − L_0_ Layer Decomposition

Given an image *I* with size M×N, the hybrid *L*_1_ − *L*_0_ layer decomposition model can be defined as follows:

(5) $$\underset{I^{B}}{\min}\sum_{i=1}^{M}\sum_{j=1}^{N}\left\{{\left(I_{i,j}^{D}\right)}^{2}+\lambda_{1}\sum_{k=\{H,\;V\}}\left|\partial_{k}I_{i,j}^{B}\right|+\lambda_{2}\sum_{k=\{H,\;V\}}F(\partial_{k}I_{i,j}^{D})\right\}$$

where $I^{B}$ and $I^{D}$ denote the base layer and the detail layer, respectively, and $I^{D}=I-I^{B}$. They are obtained by the *L*_1_ gradient sparsity term $\left|\partial_{k}I_{i,j}^{B}\right|$ and the *L*_0_ gradient sparsity term $F(\partial_{k}I_{i,j}^{D})$ accordingly. $\partial_{k}$ refers to the partial derivative operation along the horizontal gradient (*H*) or the vertical gradient (*V*). *F* is an indicator function, which is defined as:

(6) $$F(t)=\left\{\begin{array}{l} 1,\;if\;t\neq 0\\ 0,\;otherwise \end{array}\right.$$

For the convenience of calculation, Equation (5) can be rewritten in matrix vector form as follows:

(7) $$\underset{b}{\min}(\frac{1}{2}||d|{|}_{2}^{2}+\lambda_{1}||\nabla b|{|}_{1}+\lambda_{2}1^{T}F(\nabla d))$$

where $b,d\in R^{MN\times 1}$ denote the concatenated vector form of $I_{B}$ and $I_{D}$, respectively. $1\in R^{2MN}$ is a vector of all ones. $\nabla ={\left[\nabla_{x}^{T},\nabla_{y}^{T}\right]}^{T}\in R^{2MN\times MN}$, where $\nabla_{x}^{T}$ and $\nabla_{y}^{T}$ represent two gradient operator matrices in the *x* and *y* directions, respectively. F(∇d) refers to a binary vector.

By means of the Lagrangian multiplier method, Equation (7) can be converted to solve the following function:

(8) $$\begin{array}{l} L(b,d,c_{1},c_{2},y_{1},y_{2})=\frac{1}{2}||d|{|}_{2}^{2}+\lambda_{1}||c_{1}|{|}_{1}+\lambda_{2}1^{T}F(c_{2})\\ \;\;\;\;\;\;\;\;+{\left(c_{1}-\nabla b\right)}^{T}y_{1}+{\left(c_{2}-\nabla d\right)}^{T}y_{2}\\ \;\;\;\;\;\;\;\;+\frac{\rho }{2}(||c_{1}-\nabla b|{|}_{2}^{2}+||c_{2}-\nabla d|{|}_{2}^{2}) \end{array}$$

where $c_{1},c_{2}\in R^{2MN}$ denotes two auxiliary variables. $y_{1},y_{2}$ represent two Lagrangian dual variables. The optimal solution is obtained by a few iterations (15 iterations in paper [38]).

**Figure 7:**
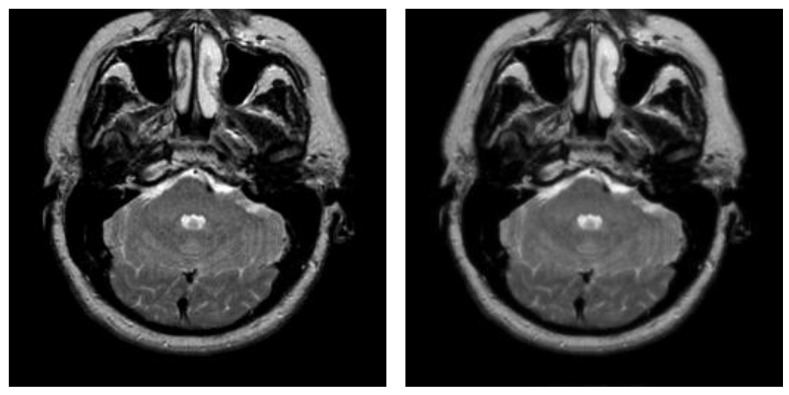

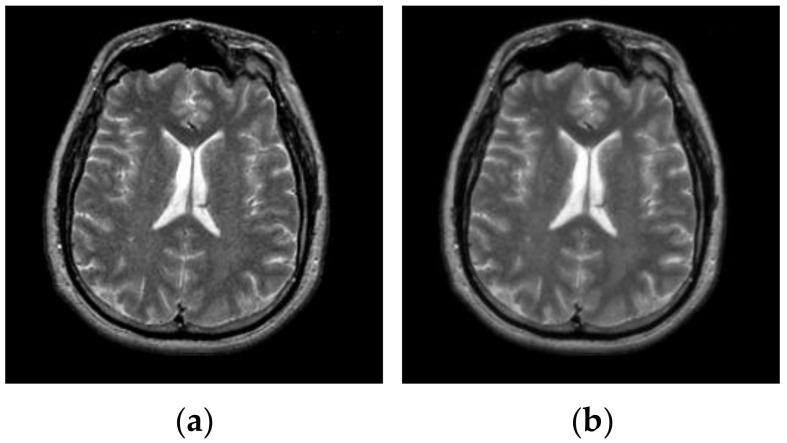
Original images and their corresponding base layers. (**a**) Original images, (**b**) base layers.

After hybrid *L*_1_ − *L*_0_ layer decomposition, the base layer of original image is used for segmentation in the framework of the proposed algorithm. Figure 7 displays an example of decomposition. In Figure 7, the first column contains two original images, and the second column contains two corresponding base layers. From Figure 7b, it can be seen that the base layers are visually smooth, and eliminate some weak edges.

### 3.3. Segmentation Fusion

A segmentation fusion method [39] is adopted to fuse different segmentation results. In the process of fusion, both spatial and intensity information is taken into account. The final segmentation result after fusion is more accurate.

Let *M*_1_, *M*_2_ represent two different segmentation maps of original image *I*, respectively. The pixels in image *I* can be grouped into two different classes by comparing *M*_1_ and *M*_2_. One is named the uncontested class, in which the class labels of the pixel in *M*_1_ and *M*_2_ are the same. The other one is named the controversial class, in which the class labels of the pixel in *M*_1_ and *M*_2_ are different. Generally, the uncontested pixels do not need to be reclassified, and the controversial pixels are considered to be misclassified and thus need to be reclassified.

Assuming that *p* is the location of a controversial pixel in image *I*, $l(p\in M_{1})=l_{a}$ and $l(p\in M_{2})=l_{b}$ denote *p*’s two different labels in *M*_1_ and *M*_2_, respectively. The reclassified class label of pixel *p* is calculated by:

(9) $$l(p)=\left\{\begin{array}{l} l_{a},\;\;\sum_{q\in {\mathbb{N}}_{p}^{r},\;l(q)=l_{a}}SIM(p,q)>\sum_{q\in {\mathbb{N}}_{p}^{r},\;l(q)=l_{b}}SIM(p,q)\\ l_{b},\;\;otherwise \end{array}\right.$$

where ${\mathbb{N}}_{p}^{r}$ denotes *p*’s effective neighborhood with radius *r*. SIM(p,q) refers to the similarity coefficient between *p* and *q*, and is defined as:

(10) $$SIM(p,q)=\frac{1}{e^{\frac{Dis(p,\;q)}{2\alpha^{2}}+\frac{\;|I(p)-I(q){|}^{2}}{2\beta^{2}}}}$$

where Dis(p, q) denotes the spatial distance between *p* and *q*. I(•) refers to the gray value of pixel •. *α* and *β* are two parameters which compromise the distance and intensity difference in constructing similarity coefficient (*α* = 1, *β* = 1 in paper [36]).

**Figure 8:**
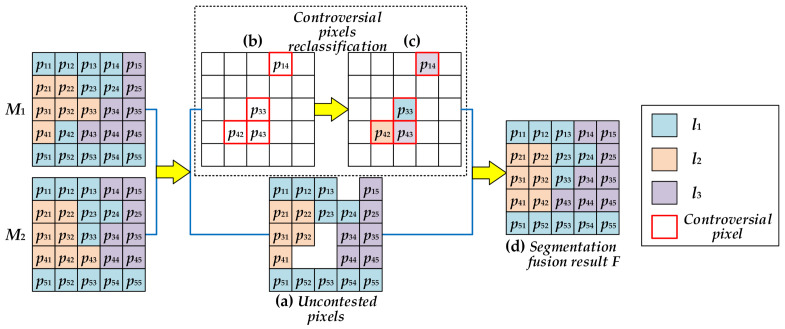
An example of segmentation fusion. *M*_1_, *M*_2_ show two different segmentation maps of an original image: (**a**) shows the uncontested pixels; (**b**) shows the controversial pixels; (**c**) shows the reclassified pixels; (**d**) shows the segmentation fusion result *F*.

Figure 8 shows a simple example of segmentation fusion. In Figure 8, it can be observed that all the pixels ${\left\{p_{ij}\right\}}_{i,j=1,\ldots ,5}$ are partitioned into three classes *l*_1_, *l*_2_, *l*_3_. The uncontested pixels are shown in Figure 8a. Pixels *p*_11_, *p*_12_, *p*_13_, *p*_23_, *p*_24_, *p*_51_, *p*_52_, *p*_53_, *p*_54_, *p*_55_ belong to class *l*_1_. Pixels *p*_21_, *p*_22_, *p*_31_, *p*_32_, *p*_41_ belong to class *l*_2_. Pixels *p*_15_, *p*_25_, *p*_34_, *p*_35_, *p*_44_, *p*_45_ belong to class *l*_3_. The remaining pixels *p*_14_, *p*_33_, *p*_42_, *p*_43_ are controversial pixels, as shown in Figure 8b. The class labels of each controversial pixel in *M*_1_ and *M*_2_ are inconsistent. Taking pixel *p*_14_ as an example, *p*_14_′s class label in map *M*_1_ is $l(p_{14}\in M_{1})=l_{1}$. However, it is classified into class *l*_3_ in map *M*_2_, i.e., $l(p_{14}\in M_{2})=l_{3}$. The four controversial pixels need to be reclassified by Equation (9). In Figure 8c, it can be seen that their final class labels are *l*(*p*_14_) = *l*_3_, *l*(*p*_33_) = *l*_2_, *l*(*p*_42_) = *l*_1_, *l*(*p*_43_) = *l*_3_. Finally, the segmentation fusion result *F* (Figure 8d) can be obtained by combining the uncontested pixels (Figure 8a) and the reclassified pixels (Figure 8c).

Segmentation maps obtained by IIMT may contain islands or isolated holes. The fusion scheme is employed to integrate the two segmentation maps to reduce misclassification pixels. It may eliminate the islands or isolated holes to obtain a better segmentation result.

## 4. Experimental Results and Analysis

### 4.1. Experimental Protocols

**Figure 9:**
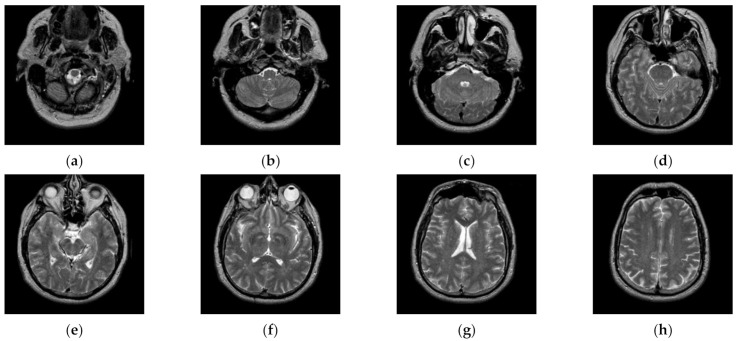

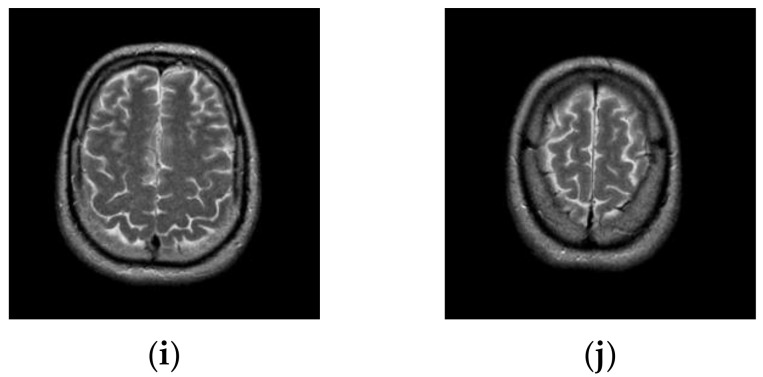
MR-T2 brain slices: (**a**) slice #022, (**b**) slice #032, (**c**) slice #042, (**d**) slice #052, (**e**) slice #062, (**f**) slice #072, (**g**) slice #082, (**h**) slice #092, (**i**) slice #102, (**j**) slice #112.

Transaxial MR-T2 brain images with various slices downloaded from “The Whole Brain Atlas” of Harvard Medical School (http://www.med.harvard.edu/aanlib/home.html, accessed on 17 May 2021) were used in the segmentation experiments. Because space is limited, the ten brain slices #022~#112 displayed in Figure 9 were chosen to demonstrate the performance of our proposed algorithm. Parameters for the proposed algorithm are listed in Table 1. All experiments were performed on a computer with Intel(R) Core(TM) i7-7500U CPU, 2.70 GHz, 8GB RAM, Windows 10 using MATLAB 8.1.0.604 (R2013a).

### 4.2. Evaluation Measure

To quantitatively evaluate the proposed algorithm and other comparison algorithms, four objective evaluation metrics were adopted in the experiments, namely, (1) uniformity measure [39,40], (2) misclassification error [7], (3) Hausdorff distance [41], and (4) Jaccard index [42].

(1) Uniformity measure

The uniformity measure can reflect the intensity difference of pixels in the same segmented class or in different segmented classes. It is defined as follows:

(11) $$U=1-2\times K\times \frac{\sum_{j=1}^{K+1}\sum_{i\in S_{j}}{\left(I_{i}-Ave(S_{j})\right)}^{2}}{M\times N\times (I_{max}-I_{min})},$$

where *K* denotes the number of thresholds; *I_i_* represents the gray value of pixel *i* in original image *I*; *S_j_* refers to the *j*th segmented class of image *I*; $Ave(S_{j})$ denotes the average gray value of all pixels in *S_j_*; *M × N* represents the size of image *I*; *I_max_* and *I_min_* denote the maximum gray value and the minimum gray value of pixels in image *I*, respectively. The values of uniformity measure *U* are between 0 and 1. The higher the value, the better the performance, and vice versa.

To fully assess the performance of the proposed algorithm, three common metrics in addition to the uniformity measure were used in the comparison experiments. Let $R_{1}$ denote the automatic segmentation of image *I*, and $R_{2}$ denote the ground-truth segmentation.

(2) Misclassification error

Misclassification error refers to the probability of pixels being misclassified, namely, the ratio of foreground pixels incorrectly classified as background pixels and background pixels incorrectly classified as foreground pixels, to all pixels. Misclassification error is defined as:

(12) $$ME=1-\frac{\left|R_{1}^{foreground}\cap R_{2}^{foreground}|+|R_{1}^{background}\cap R_{2}^{background}\right|}{\left|R_{2}^{foreground}|+|R_{2}^{background}\right|}$$

where $R_{1}^{foreground}$ and $R_{1}^{background}$ denote the foreground region and background region of *R*_1_, respectively; $R_{2}^{foreground}$ and $R_{2}^{background}$ denote the foreground region and background region of *R*_2_, respectively.

(3) Hausdorff distance

The Hausdorff distance is defined as:

(13) $$H(R_{1},R_{2})=\max\{h(R_{1},R_{2}),h(R_{2},R_{1})\}$$

where $h(R_{1},R_{2})=\underset{a_{i}\in R_{1}}{\max}\underset{b_{j}\in R_{2}}{\min}||a_{i}-b_{j}||$ and $h(R_{2},R_{1})=\underset{b_{j}\in R_{2}}{\max}\underset{a_{i}\in R_{1}}{\min}||b_{j}-a_{i}||$. A higher Hausdorff distance indicates a larger difference between the two segmentations $R_{1}$ and $R_{2}$. Hence, a satisfactory segmentation corresponds to a low Hausdorff distance.

(4) Jaccard index

The Jaccard index is defined as:

(14) $$J(R_{1},R_{2})=\frac{\left|R_{1}\cap R_{2}\right|}{\left|R_{1}\cup R_{2}\right|}$$

The value of Jaccard index varies from 0 to 1. Higher values of *J* indicate better segmentation.

### 4.3. Comparison with Otsu-Based Method

In this paper, the newly proposed segmentation algorithm (subsequently referred to as “Proposed”) is based on the Otsu method. To verify its effectiveness, this subsection compares it with three Otsu-based algorithms in terms of single thresholding (*K* = 1) and multilevel thresholding (*K* = 2, 3, 4, 5). The comparison algorithms include (1) the original Otsu method (Otsu), (2) the newly proposed interval iteration multilevel thresholding method (IIMT), and (3) IIMT based on Hybrid *L*_1_ − *L*_0_ layer decomposition (HL-IIMT).

**Figure 10:**
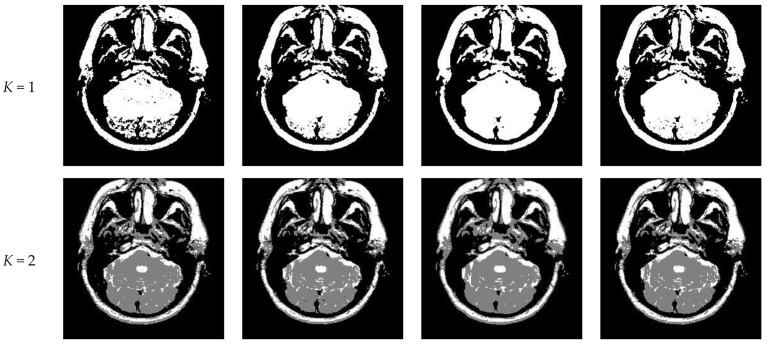

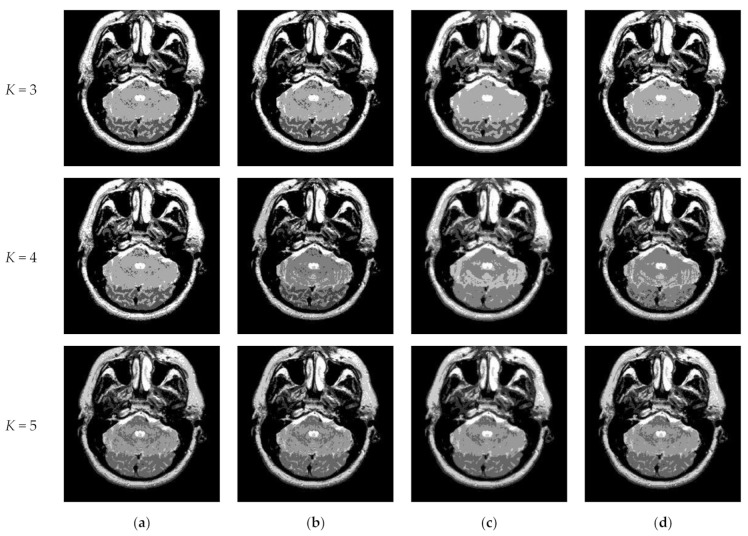
Segmentation results obtained by different segmentation algorithms for slice #042 with number of thresholds *K* from 1 to 5: (**a**) Otsu, (**b**) IIMT, (**c**) HL-IIMT, (**d**) Proposed.

**Figure 11:**
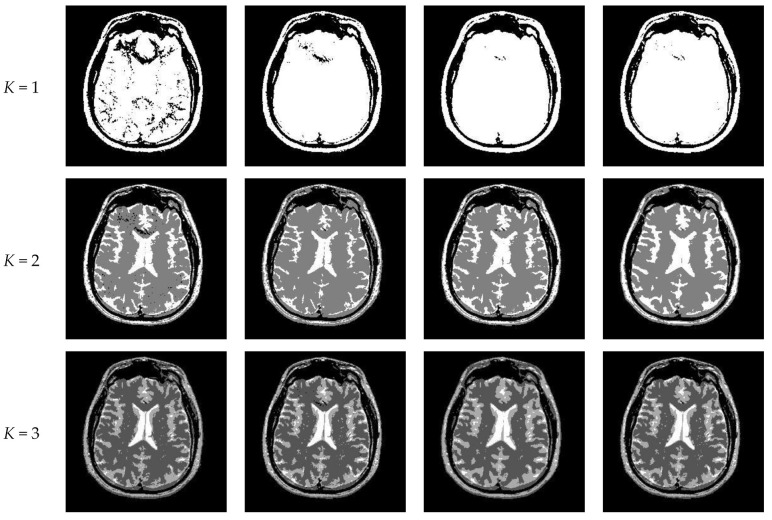

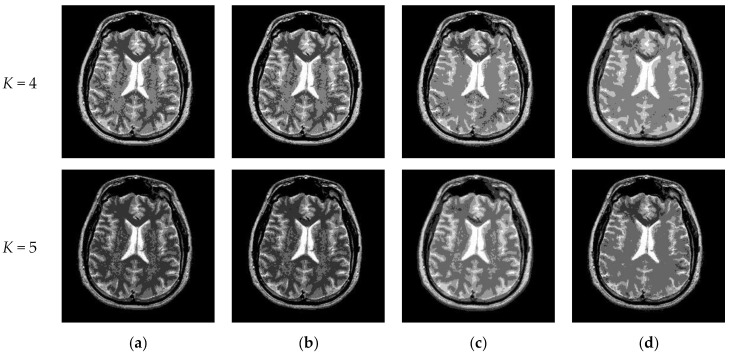
Segmentation results obtained by different segmentation algorithms for slice #082 with number of thresholds *K* from 1 to 5: (**a**) Otsu, (**b**) IIMT, (**c**) HL-IIMT, (**d**) Proposed.

Figure 10 and Figure 11 display segmentation results of different algorithms for slice #042 and slice #082, respectively. For single level of thresholding *K* = 1, it can be observed that segmentation results obtained by the Otsu method have many fragmented small areas, such as the lower soft tissue in the first row of Figure 10a, whereas IIMT performs slightly better. However, the edges segmented by HL-IIMT and Proposed are much clearer. In the case of K≥2, it can be seen that Otsu and IIMT have similar segmentation effects. HL-IIMT and Proposed are better than Otsu and IIMT in terms of edge-preserving and denoising, as shown in the segmentation results in Figure 11 (*K* = 2, *K* = 4).

**Figure 12:**
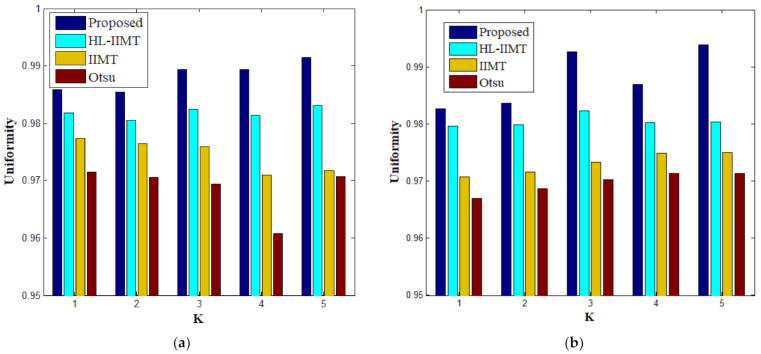
Values of the uniformity measure for different segmentation algorithms with number of thresholds *K* from 1 to 5. (**a**) #042, (**b**) #082.

Table 2 shows the values of uniformity measure (*U*) of Proposed, HL-IIMT, IIMT, and Otsu algorithms for slice #042 and slice #082. The best evaluation results are marked in bold. It can be noted that the *U* values achieved by Proposed are the highest for both of the two test images. To more clearly present the results, Figure 12 illustrates the comparison of *U* for different algorithms based on Table 2. In Figure 12, it can be clearly noted that Proposed achieves the highest values, and HL-IIMT comes second, followed by IIMT and Otsu. This indicates that the novel thresholding method IIMT presented in this paper is effective, and our Proposed based on IIMT can obtain satisfactory segmentation results with clear edges and little noise.

### 4.4. Experimental Results on Images Containing Noise

**Figure 13:**
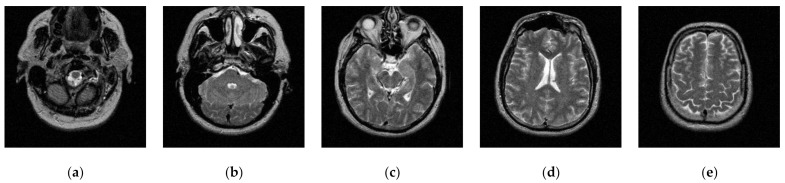
Images containing noise via the addition of Gaussian noise *N* (0, 0.001): (**a**) slice #022, (**b**) slice #042, (**c**) slice #062, (**d**) slice #082, (**e**) slice #102.

This subsection compares segmentation results of different algorithms (Proposed, Otsu, IIMT, and HL-IIMT) on images containing noise. Figure 13 displays five images with Gaussian noise *N* (0, 0.001) added to images #022, #042, #062, #082, and #102, which were selected from Figure 9.

**Figure 14:**
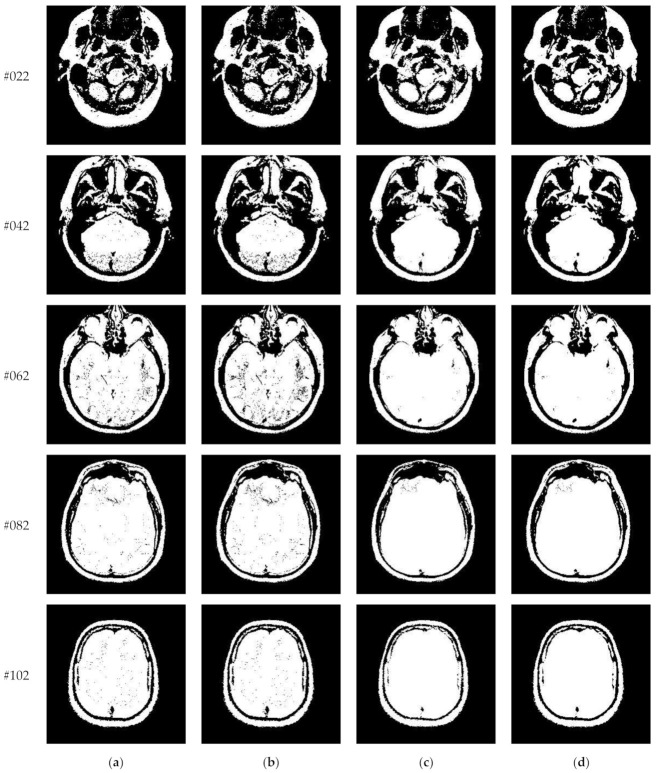
Segmentation results obtained by different segmentation algorithms for images containing noise (*K* = 1): (**a**) Otsu, (**b**) IIMT, (**c**) HL-IIMT, (**d**) Proposed.

**Figure 15:**
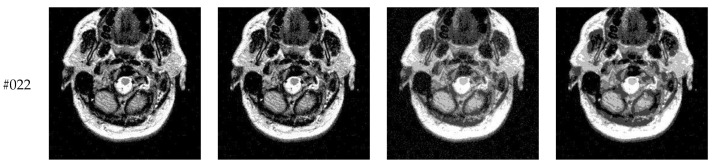

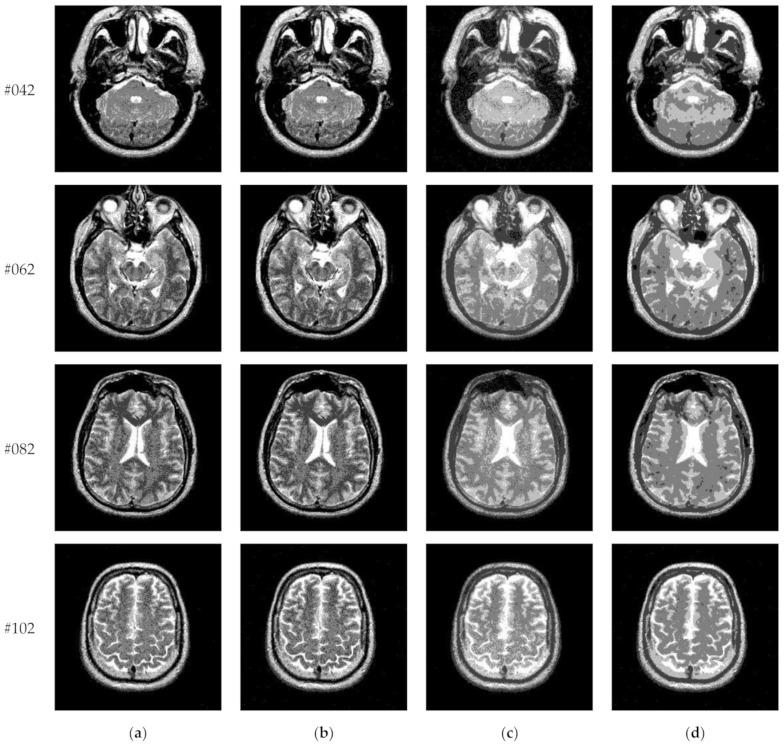
Segmentation results obtained by different segmentation algorithms for images containing noise (*K* = 4): (**a**) Otsu, (**b**) IIMT, (**c**) HL-IIMT, (**d**) Proposed.

Figure 14 displays the segmentation results of images containing noise with a single level of thresholding *K* = 1. It can be observed that segmentation results achieved by HL-IIMT and Proposed are distinctly better than those of Otsu and IIMT, which have many isolated points. Figure 15 depicts segmentation results obtained by different algorithms with multilevel thresholding *K* = 4. Obviously, segmentation results of Otsu, IIMT, and HL-IIMT are seriously affected by noise, and most regions are blurred. However, the results of Proposed are better, and they have less noise and clearer edges.

**Figure 16:**
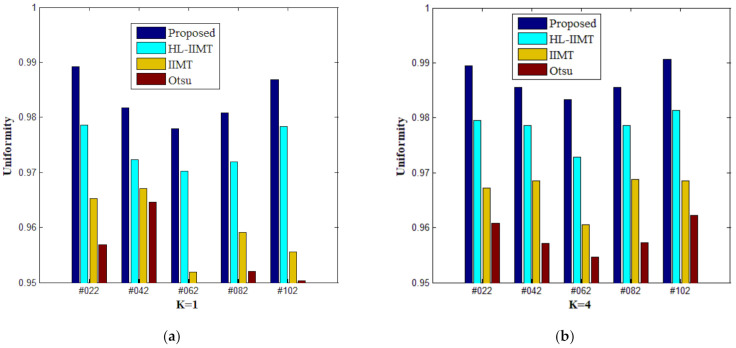
Values of the uniformity measure for different segmentation algorithms with number of thresholds *K* = 1, 4: (**a**) *K* = 1, (**b**) *K* = 4.

A comparison of the evaluation results for different segmentation algorithms on images containing noise with *K* = 1, 4 is shown in Table 3, and corresponding comparison charts are given in Figure 16. In Table 3, the best results are marked in bold. It can be noted that Proposed consistently has the highest *U* values. For images containing noise, both the IIMT-based algorithms (HL-IIMT and Proposed) are superior to the original Otsu method in single threshold segmentation; furthermore, Proposed can achieve satisfactory results in multilevel threshold segmentation compared to the other three algorithms (IIMT, HL-IIMT, and Otsu).

### 4.5. Comprehensive Comparison

To comprehensively evaluate the performance of our proposed algorithm, segmentation results of “Proposed” were compared with those of six other multilevel thresholding algorithms in this experiment, namely, the local Laplacian filtering and discrete curve evolution-based method (LLF-DCE) [39], the particle swarm optimization-based method (PSO), the bacterial foraging-based method (BF) and adaptive bacterial foraging-based method (ABF) [43], the Nelder–Mead simplex-based method (NMS), and the real coded genetic algorithm (RCGA) [40]. Brief descriptions of the eight algorithms are as follows.

(1) Proposed

In the proposed algorithm, the initial thresholds and mean value of each class are obtained by Otsu multilevel thresholding. Then, Otsu single thresholding is iteratively performed on each interval to search for the optimal threshold in the sub-region.

(2) LLF-DCE

In LLF-DCE method, discrete curve evolution (DCE) is used to simplify the curve shape of the image histogram, and important points are reserved that are generally in peak or valley regions [39]. Gray levels corresponding to these points comprise a series of intervals. Then, Otsu single thresholding is performed in each interval to search for the optimal threshold.

(3) PSO

PSO is a stochastic global optimization algorithm and simulates the foraging behavior of birds. The bird is simulated by a massless particle which has two attributes: speed and position. The optimal solution can be sought by continuously updating the speed and position.

(4) BF

BF is a heuristic algorithm. In the process of maximizing Kapur’s entropy and between-class variance, BF is adopted to search for optimal thresholds by simulating the foraging behavior of *Escherichia coli* in the human gut. The behavior specifically includes four actions: chemotaxis, swarming, reproduction, and elimination-dispersal.

(5) ABF

In the ABF method, an adaptive step size is employed in the traditional bacterial foraging method to improve the exploration and exploitation capability.

(6) NMS

NMS is a direct search method for multi-dimensional unconstrained minimization. NMS is used to optimize maximum entropy method to identify optimum thresholds.

(7) RCGA

In the RCGA method, simulated binary crossover (SBX) is employed in crossover and mutation mechanisms of a real coded genetic algorithm. SBX is essentially adaptive, and it creates child solutions proportionally based on the difference in parent solutions. Then, the optimal thresholds are found by maximizing Kapur’s entropy.

**Figure 17:**
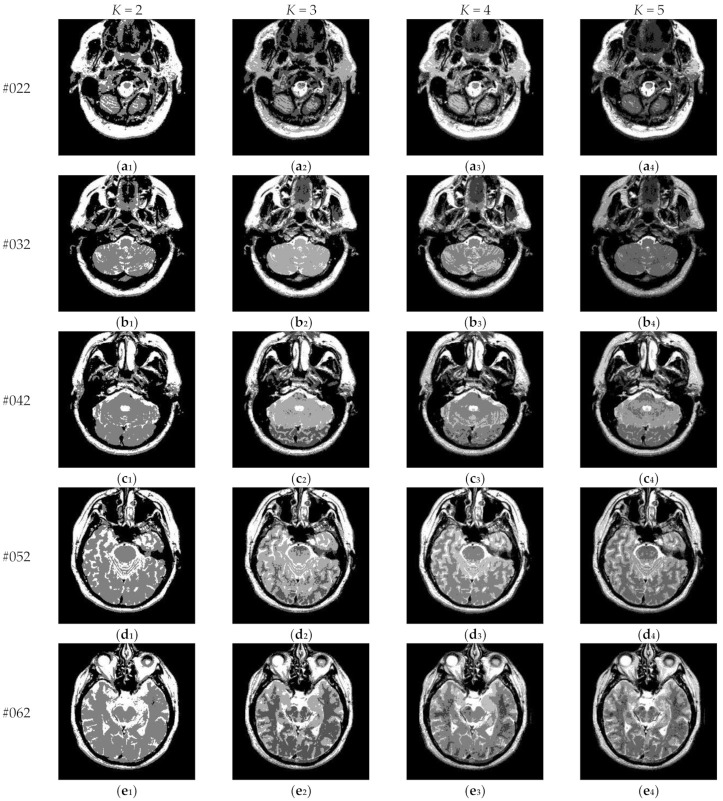

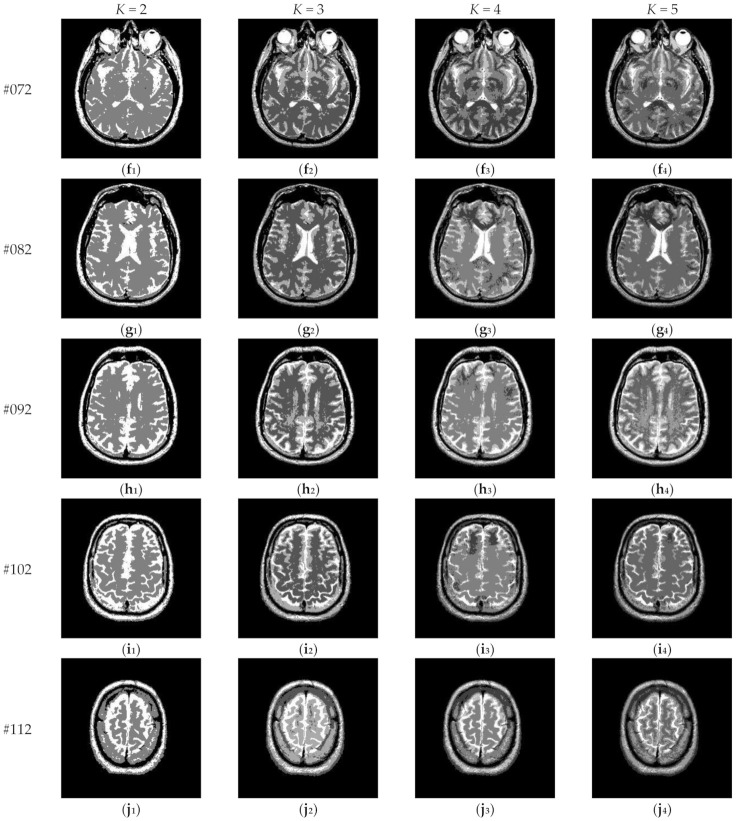
Segmentation results obtained by the proposed algorithm for brain slices #022~#112: (**a_1_**–**j_1_**) display the results of 2-thresholding; (**a_2_**–**j_2_**) display the results of 3-thresholding; (**a_3_**–**j_3_**) display the results of 4-thresholding; (**a_4_**–**j_4_**) display the results of 5-thresholding.

Figure 17 depicts the segmentation results of Proposed for brain slices #022~#112 with the number of thresholds *K* from 2 to 5. It can be seen that segmentation results with different threshold numbers have different effects. In general, the higher the level of thresholding, the better segmentation quality. Table 4 displays the comparison of optimal threshold values obtained by different algorithms with *K* = 2, 3, 4, 5. The proposed algorithm and LLF-DCE are based on the fusion scheme. The former combines two different segmentation results obtained by IIMT and HL-IIMT; the latter combines two different segmentation results obtained by LLF-Otsu and DCE-Otsu. In Table 4, it can be seen that the final thresholds selected by different algorithms are different from each other.

Table 5 shows the uniformity measure (*U*) values of different segmentation algorithms. The best results are marked in bold. It is clear that the *U* value of Proposed is the highest for each test image and each level of thresholding. The proposed algorithm is superior to PSO, BF, ABF, NMS, and RGA in most cases. Taking test image #062 as an example, in the case of *K* = 2 and 4, *U* values of Proposed are more than 0.98, whereas the best evaluation result of the above five algorithms is merely 0.9236 (PSO, *K* = 4). For *K* = 3 and 5, *U* values of Proposed are more than 0.99, whereas the best results obtained by PSO, BF, ABF, NMS and RGA are 0.9835 (NMS, *K* = 5) and 0.9855 (RGA, *K* = 5), and the remainder are all below 0.95. Compared to the DCE method, the evaluation values of Proposed and LLF-DCE are not significantly different, and Proposed performs slightly better for each test image.

**Figure 18:**
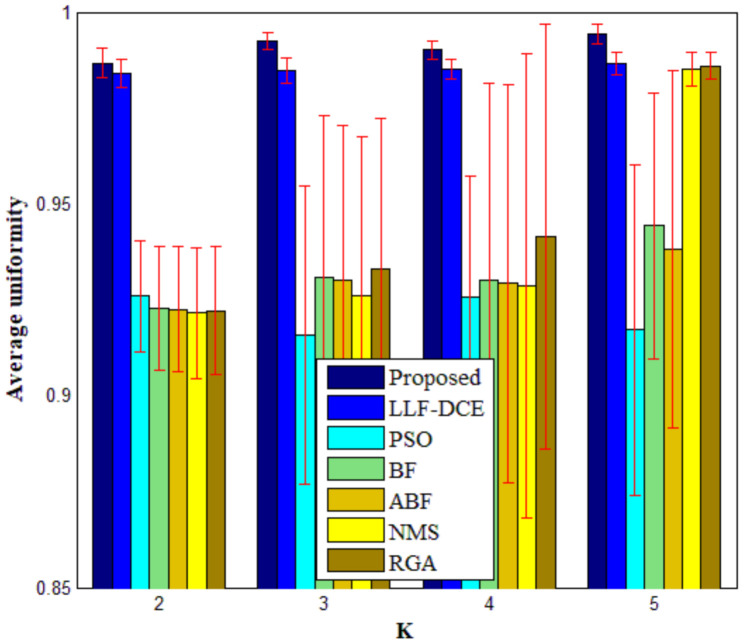
Average values and standard deviations of the uniformity measure for different segmentation algorithms with number of thresholds *K* from 2 to 5.

**Figure 19:**
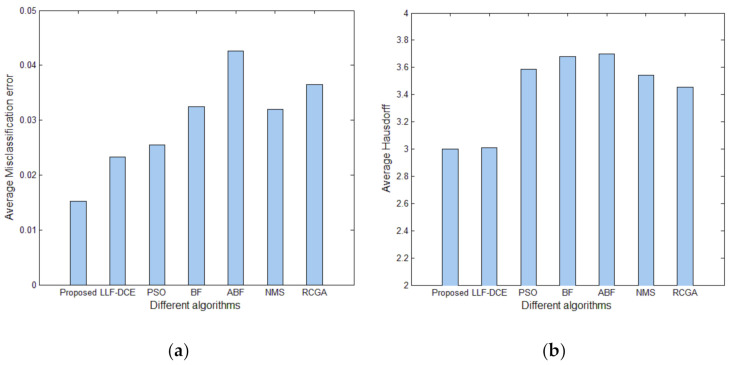

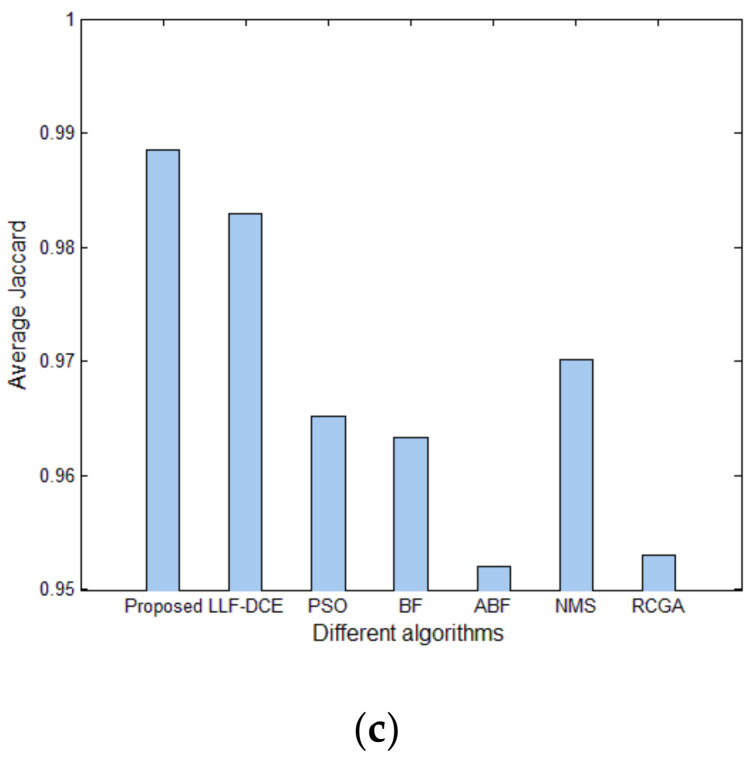
Comparison of evaluation results for different algorithms: (**a**) average misclassification error, (**b**) average Hausdorff distance, (**c**) average Jaccard index.

In order to show the comprehensive performance of the proposed algorithm, Figure 18 shows average values and standard deviations of *U* for different segmentation algorithms with the number of thresholds *K* from 2 to 5. It can be noted that the average *U* values of the proposed algorithm are higher than those of other comparison algorithms for each level of thresholding, which indicates superior segmentation quality. In particular, they are significantly higher than the average *U* values of PSO, BF, ABF, NMS, and RGA in the cases of *K* = 2, 3, 4. The error bars (standard deviations) of Proposed and LLF-DCE are obviously shorter than those of other segmentation algorithms. Figure 19 shows the comparison of average values of the misclassification error, Hausdorff distance, and Jaccard index for different algorithms. It can be noted that the proposed algorithm achieves the lowest misclassification error and Hausdorff distance, and the highest Jaccard index. In addition, LLF-DCE also performs well when compared with others.

In summary, our proposed algorithm performs better than other comparison segmentation algorithms. It can not only achieve good segmentation results but also has excellent stability.

### 4.6. Experimental Results on BRATS Database

**Figure 20:**
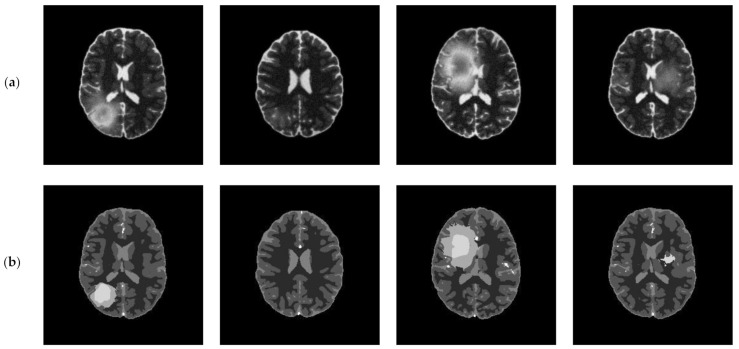
An example of original images and ground truth from BRATS: (**a**) original images, (**b**) ground truth.

In this subsection, we applied the proposed algorithm to the BRATS (Multimodal Brain Tumor Image Segmentation Benchmark) database. The BRATS database (http://www.imm.dtu.dk/projects/BRATS2012/data.html, accessed on 25 September 2021) is compiled from the international brain tumor segmentation challenge in MICCAI 2012 conference. It is a widely used database and composed of multi-contrast brain MR scans of 25 low-grade and 25 high-grade glioma cases and the corresponding ground truth. Each case includes four modalities—T1, T1c, T2, and FLAIR [44]—and each MR scanning sequence contains more than one hundred images. Figure 20 presents an example of brain MR images from BRATS. Figure 20a shows the original images and the corresponding ground truth is displayed in Figure 20b.

**Figure 21:**
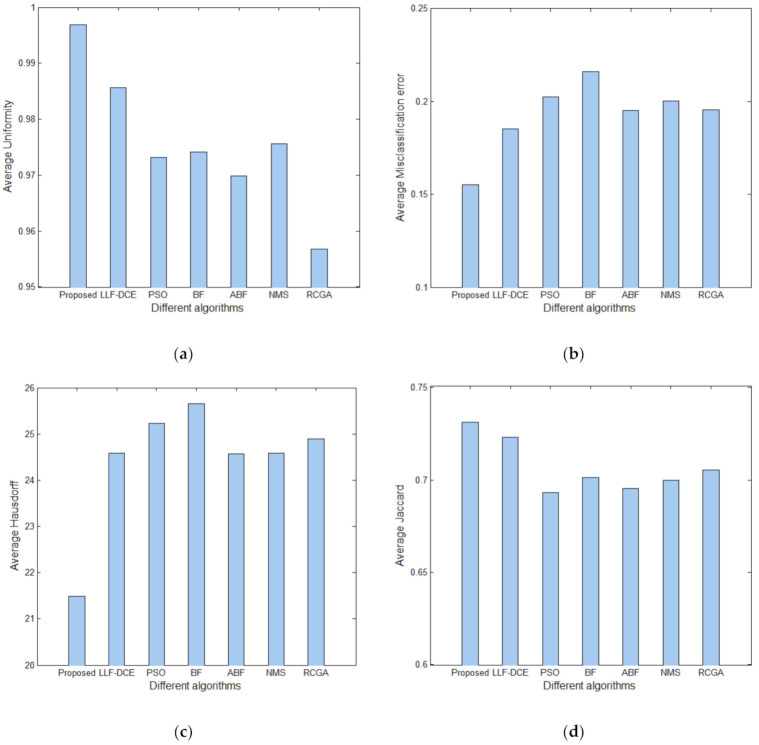
Comparison of evaluation results for different algorithms: (**a**) average uniformity measure, (**b**) average misclassification error, (**c**) average Hausdorff distance, (**d**) average Jaccard index.

The performance of the proposed algorithm on BRATS was compared with other segmentation algorithms in terms of the uniformity measure, misclassification error, Hausdorff distance, and Jaccard index. Figure 21 shows the average evaluation values for different algorithms. It can be observed that the proposed algorithm achieves excellent results in terms of the uniformity measure and Hausdorff distance, as shown in Figure 21a,c, which are obviously better than those of other algorithms. From Figure 21b,d, the proposed algorithm also performs best, followed by LL-DCE.

## 5. Conclusions

In this paper, a novel multilevel thresholding algorithm based on interval iteration (named IIMT) for brain MR images is proposed. In contrast to most other multilevel thresholding methods, IIMT iteratively searches for sub-regions of the image to achieve segmentation, rather than taking the original image as a whole. First, standard Otsu multilevel thresholding is performed on the original image to obtain initial thresholds and class means. Then, in the succeeding iteration, standard Otsu single thresholding is used to determine the threshold in each interval formed by the class means derived in the previous iteration. For two adjacent peaks in the gray histogram, the optimal threshold is found if the difference between thresholds obtained in two consecutive iterations is less than a preset value. Iterating is stopped when all optimal thresholds are found. Furthermore, we presented an IIMT-based segmentation framework for brain MR images. The hybrid *L*_1_ − *L*_0_ layer decomposition method is utilized to decompose the original image to derive its base layer. IIMT is separately performed on the original image and its base layer to gain two different segmentation results. In order to improve the segmentation accuracy, a fusion scheme is adopted to fuse these two results. Experimental results verified that the proposed algorithm is applicable and can achieve satisfactory segmentation results. Compared to other multilevel thresholding algorithms, the proposed algorithm can obtain a better visual effect and, subjectively, its segmentation results have clear edges and little noise. The uniformity measure, misclassification error, Hausdorff distance, and Jaccard index objectively demonstrated the performance of the proposed algorithm. The proposed algorithm results in effective segmentation for medical images, and shows excellent stability and robustness for images containing noise. In clinical medicine, the proposed algorithm can assist doctors to diagnose diseases, locate the lesion area, and detect changes in tumor volume and size. It also can be used in pre-processing for other image processing technologies, such as image fusion.

In future, our research work can be extended in three directions. First, the design idea of determining thresholds in the proposed IIMT can be incorporated into other multilevel thresholding algorithms and extended into 2D/3D Otsu or similar methods, such as maximum entropy and minimum error. Second, more effective segmentation fusion strategies can be designed to improve the quality of medical image segmentation. Finally, deep convolutional neural networks can be adopted to image segmentation. We will combine traditional image segmentation techniques with deep learning models to with the aim of achieving good segmentation effects.

## Figures and Tables

**Table 1:** Parameter settings of the proposed algorithm.

Parameter Settings	Description
*δ* = 0.01	Value that stops the iteration for IIMT
*λ*_1_ = 1	Weight of base layer for hybrid *L*_1_ − *L*_0_ layer decomposition
*λ*_2_ = 0.1*λ*_1_	Weight of detail layer for hybrid *L*_1_ − *L*_0_ layer decomposition
*r* = 12	Radius for segmentation fusion
*K* = 1, 2, 3, 4, 5	Number of the thresholds

**Table 2:** Comparison of uniformity measure for different segmentation algorithms.

Test Images	Number of Thresholds (*K*)	Uniformity Measure (*U*)			
		Proposed	HL-IIMT	IIMT	OTSU
#042	1	**0.9858**	0.9818	0.9773	0.9715
	2	**0.9855**	0.9805	0.9764	0.9705
	3	**0.9893**	0.9825	0.9759	0.9694
	4	**0.9893**	0.9814	0.9709	0.9608
	5	**0.9914**	0.9831	0.9717	0.9707
#082	1	**0.9827**	0.9796	0.9708	0.9670
	2	**0.9836**	0.9799	0.9716	0.9687
	3	**0.9927**	0.9823	0.9733	0.9702
	4	**0.9869**	0.9802	0.9749	0.9713
	5	**0.9938**	0.9804	0.9750	0.9714

**Table 3:** Comparison of uniformity measure for different segmentation algorithms on images containing noise.

Test Images	Number of Thresholds (*K*)	Uniformity Measure (*U*)			
		Proposed	HL-IIMT	IIMT	OTSU
#022	1	**0.9892**	0.9786	0.9652	0.9569
	4	**0.9895**	0.9795	0.9672	0.9608
#042	1	**0.9817**	0.9723	0.9671	0.9646
	4	**0.9856**	0.9786	0.9685	0.9571
#062	1	**0.9780**	0.9702	0.9519	0.9407
	4	**0.9833**	0.9728	0.9605	0.9547
#082	1	**0.9808**	0.9719	0.9591	0.9520
	4	**0.9856**	0.9786	0.9688	0.9572
#102	1	**0.9869**	0.9784	0.9556	0.9503
	4	**0.9906**	0.9813	0.9685	0.9622

**Table 4:** Comparison of optimal threshold values obtained by applying different segmentation algorithms to the test images.

Test Images	*K*	Optimal Threshold Values								
		Proposed		LLF-DCE		PSO	BF	ABF	NMS	RCGA
		IIMT	HL-IIMT	LLF-Otsu	DCE-Otsu					
#022	2	40, 96	34, 103	26, 95	1, 77	97, 184	96, 184	95, 184	96, 184	96, 184
	3	42, 98, 156	22, 69, 125	26, 64, 103	1, 3, 77	69, 138, 207	65, 131, 186	69, 114, 185	58, 116, 185	58, 115, 185
	4	20, 48, 86, 126	20, 60, 100, 141	26, 64, 91, 132	1, 3, 5, 79	83, 116, 175, 207	52, 99, 148, 186	58, 113, 174, 208	43, 87, 132, 185	44, 87, 131, 186
	5	28, 70, 118, 164, 228	17, 51, 90, 132, 178	14, 26, 64, 91, 132	1, 3, 5, 68, 79	76, 119, 154, 184, 214	44, 90, 127, 170, 208	43, 88, 130, 176, 208	44, 104, 140, 176, 214	44, 86, 127, 174, 208
#032	2	50, 112	43, 115	25, 102	1, 77	107, 185	110, 185	110, 185	110, 185	109, 185
	3	28, 70, 120	22, 73, 124	25, 82, 110	1, 3, 79	74, 157, 192	72, 120, 198	81, 134, 187	56, 115, 186	53, 116, 185
	4	24, 66, 112, 152	22, 73, 124, 181	25, 82, 94, 151	1, 3, 5, 81	95, 125, 164, 194	63, 119, 173, 208	58, 102, 142, 190	39, 83, 132, 189	39, 84, 131, 189
	5	30, 74, 118, 158, 204	15, 47, 81, 112, 148	25, 56, 88, 97, 151	1, 3, 5, 57, 81	80, 112, 139, 186, 213	63, 101, 140, 175, 207	52, 87, 128, 167, 198	29, 75, 124, 173, 207	34, 78, 123, 174, 207
#042	2	54, 118	46, 120	29, 111	37, 87	111, 183	114, 184	114, 184	113, 184	114, 183
	3	34, 82, 130	27, 82, 130	29, 69, 132	37, 49, 141	80, 148, 178	70, 136, 188	74, 130, 185	84, 132, 188	84, 132, 187
	4	36, 76, 112, 156	21, 67, 105, 149	29, 69, 97, 144	37, 48, 95, 143	81, 125, 164, 197	62, 112, 156, 194	50, 100, 143, 190	29, 76, 128.187	30, 75, 127, 188
	5	20, 56, 90, 126, 168	18, 60, 94, 128, 170	29, 69, 78, 108, 145	35, 49, 77, 95, 143	82, 115, 142, 184, 214	58, 114, 151, 188, 218	53, 97, 144, 184, 218	31, 76, 126, 178, 217	25, 69, 114, 156, 194
#052	2	58, 114	49, 111	30, 103	31, 89	119, 186	117, 186	117, 186	118, 185	118, 185
	3	46, 88, 130	31, 88, 127	30, 75, 111	31, 45, 125	89, 113, 187	102, 156, 206	107, 158, 204	109, 166, 207	109, 165, 203
	4	22, 60, 96, 134	19, 65, 99, 135	30, 75, 93, 143	31, 45, 79, 127	79, 111, 141, 208	93, 124, 171, 210	90, 129, 173, 210	94, 132, 175, 210	91, 131, 174, 209
	5	22, 58, 88, 120, 156	35, 89, 120, 152, 194	14, 30, 75, 93, 143	31, 45, 79, 100, 127	65, 85, 131, 162, 203	56, 112, 144, 175, 209	56, 95, 133, 167, 203	20, 67, 120, 167, 207	24, 67, 118, 166, 203
#062	2	58, 120	51, 118	31, 111	33, 103	109, 186	119, 190	119, 186	121, 187	121, 187
	3	48, 94, 144	42, 97, 142	31, 79, 134	33, 45, 133	112, 167, 187	97, 133, 183	102, 147, 199	101, 148, 195	101, 147, 196
	4	42, 84, 120, 164	19, 67, 106, 149	31, 79, 96, 151	33, 45, 81, 135	85, 134, 180, 203	98, 140, 182, 218	93, 135, 175, 212	94, 134, 176, 211	94, 134, 175, 211
	5	28, 64, 94, 128, 170	27, 75, 102, 137, 179	17, 31, 79, 96, 151	33, 45, 81, 116, 135	99, 119, 157, 181, 203	73, 104, 139, 184, 213	79, 111, 145, 179, 212	28, 68, 120, 168, 208	20, 65, 113, 158, 200
#072	2	60, 120	52, 120	32, 133	33, 111	116, 177	117, 179	117, 179	118, 179	117, 179
	3	54, 100, 156	47, 103, 156	32, 76, 139	33, 45, 139	96, 178, 207	95, 147, 202	99, 150, 190	100, 142, 188	99, 141, 187
	4	48, 86, 122, 178	36, 87, 122, 174	32, 76, 93, 155	33, 45, 81, 141	96, 124, 161, 187	94, 129, 173, 214	95, 134, 174, 214	100, 140, 179, 214	99, 140, 179, 213
	5	48, 84, 110, 142, 188	17, 62, 94, 128, 179	32, 68, 81, 102, 155	33, 45, 63, 81, 141	72, 112, 151, 178, 197	87, 109, 139, 178, 210	87, 119, 150, 180, 214	10, 64, 120, 172, 211	14, 64, 119, 171, 211
#082	2	60, 116	51, 113	32, 110	37, 103	110, 170	112, 169	111, 170	112, 169	111, 169
	3	54, 102, 158	47, 102, 158	32, 83, 143	37, 49, 137	103, 136, 198	114, 155, 210	111, 155, 201	103, 146, 189	103, 146, 190
	4	42, 82, 116, 168	20, 70, 105, 158	32, 83, 93, 166	37, 49, 87, 138	100, 129, 167, 188	103, 139, 175, 214	99, 135, 170, 210	98, 134, 169, 210	98, 133, 169, 210
	5	52, 88, 118, 154, 210	17, 63, 92, 121, 171	15, 32, 83, 93, 166	37, 49, 87, 99, 139	78, 105, 151, 180, 201	81, 122, 150, 182, 212	84, 113, 146, 178, 214	14, 62, 115, 168, 210	10, 62, 107, 148, 190
#092	2	58, 108	55, 115	33, 104	35, 101	109, 175	108, 174	109, 174	109, 173	109, 174
	3	52, 92, 134	46, 97, 135	33, 78, 109	35, 47, 123	115, 134, 178	107, 144, 209	104, 158, 207	106, 158, 206	105, 158, 206
	4	40, 78, 106, 144	19, 70, 105, 143	33, 78, 93, 143	35, 47, 81, 125	77, 107, 149, 194	100, 129, 164, 208	102, 138, 171, 212	112, 152, 186, 220	97, 136, 211, 173
	5	24, 60, 84, 110, 148	18, 65, 94, 120, 154	33, 66, 83, 104, 143	35, 47, 81, 92, 125	90, 113, 165, 185, 206	85, 114, 147, 175, 212	96, 128, 158, 186, 216	10, 64, 110, 160, 205	5, 62, 109, 159, 205
#102	2	56, 108	53, 114	31, 102	33, 99	98, 166	108, 174	108, 174	108, 173	107, 174
	3	50, 92, 136	45, 100, 144	31, 66, 108	33, 47, 127	113, 145, 180	103, 148, 189	98, 146, 189	94, 142, 189	94, 142, 190
	4	56, 96, 138, 184	20, 70, 106, 147	31, 66, 94, 143	33, 45, 79, 127	84, 124, 165, 189	79, 122, 164, 200	90, 127, 164, 198	2, 64, 119, 173	1, 63, 120, 174
	5	50, 84, 114, 146, 182	19, 67, 97, 125, 158	31, 61, 79, 100, 143	31, 45, 60, 79, 127	99, 128, 147, 194, 218	81, 113, 147, 187, 220	82, 114, 148, 184, 218	9, 62, 106, 147, 190	1, 62, 104, 145, 189
#112	2	54, 106	48, 121	25, 96	35, 81	109, 162	105, 165	105, 164	106, 163	106, 163
	3	34, 78, 122	28, 87, 138	25, 78, 106	35, 51, 137	104, 163, 216	79, 134, 180	71, 123, 175	3, 49, 145	1, 70, 142
	4	40, 74, 106, 148	25, 79, 119, 164	25, 71, 89, 148	35, 51, 91, 139	63, 130, 153, 206	54, 117, 156, 192	58, 105, 146, 182	4, 63, 132, 178	1, 65, 123, 172
	5	28, 66, 100, 144, 194	21, 64, 100, 129, 170	25, 49, 84, 94, 148	35, 51, 91, 93, 141	58, 128, 155, 187, 213	48, 112, 137, 161, 200	47, 108, 142, 171, 197	2, 44, 79, 131, 175	1, 49, 95, 139, 183

**Table 5:** Comparison of the uniformity measure for different segmentation algorithms.

Test Images	Number of Thresholds (*K*)	Uniformity Measure (*U*)						
		Proposed	DCE	PSO	BF	ABF	NMS	RCGA
#022	2	**0.9879**	0.9860	0.9552	0.9569	0.9569	0.9569	0.9569
	3	**0.9956**	0.9795	0.9672	0.9708	0.9696	0.9769	0.9769
	4	**0.9912**	0.9847	0.9420	0.9765	0.9698	0.9824	0.9824
	5	**0.9975**	0.9837	0.9435	0.9786	0.9785	0.9752	0.9788
#032	2	**0.9894**	0.9844	0.9368	0.9342	0.9342	0.9342	0.9342
	3	**0.9910**	0.9863	0.9619	0.9716	0.9600	0.9796	0.9801
	4	**0.9920**	0.9855	0.9144	0.9697	0.9766	0.9848	0.9848
	5	**0.9983**	0.9852	0.9422	0.9668	0.9767	0.9851	0.9843
#042	2	**0.9855**	0.9823	0.9271	0.9246	0.9246	0.9246	0.9246
	3	**0.9893**	0.9826	0.9585	0.9721	0.9689	0.9548	0.9548
	4	**0.9893**	0.9853	0.9465	0.9752	0.9821	0.9865	0.9865
	5	**0.9914**	0.9893	0.9348	0.9724	0.9766	0.9845	0.9877
#052	2	**0.9882**	0.9840	0.9158	0.9128	0.9128	0.9068	0.9128
	3	**0.9907**	0.9849	0.9523	0.9713	0.9673	0.8800	0.9467
	4	**0.9892**	0.9861	0.9372	0.9764	0.9834	0.8982	0.9856
	5	**0.9933**	0.9875	0.9240	0.9735	0.9782	0.9842	0.9868
#062	2	**0.9818**	0.9802	0.9192	0.9047	0.9049	0.9015	0.9015
	3	**0.9906**	0.9823	0.8777	0.9135	0.9029	0.9030	0.9030
	4	**0.9868**	0.9805	0.9236	0.8856	0.8988	0.8989	0.8989
	5	**0.9907**	0.9828	0.8505	0.9527	0.9325	0.9835	0.9855
#072	2	**0.9799**	0.9786	0.9068	0.9041	0.9041	0.9041	0.9041
	3	**0.9910**	0.9821	0.9034	0.9084	0.8985	0.8992	0.8992
	4	**0.9890**	0.9830	0.8809	0.8876	0.8804	0.8666	0.8666
	5	**0.9917**	0.9830	0.9531	0.8881	0.8876	0.9818	0.9825
#082	2	**0.9836**	0.9791	0.9120	0.9091	0.9091	0.9091	0.9091
	3	**0.9927**	0.9837	0.8852	0.8621	0.8661	0.8849	0.8849
	4	**0.9869**	0.9830	0.8619	0.8479	0.8622	0.8695	0.8695
	5	**0.9938**	0.9860	0.9372	0.9188	0.9105	0.9854	0.9857
#092	2	**0.9893**	0.9887	0.9131	0.9156	0.9131	0.9131	0.9131
	3	**0.9948**	0.9890	0.8607	0.8751	0.8827	0.8786	0.8786
	4	**0.9904**	0.9865	0.9490	0.8583	0.8514	0.8240	0.8641
	5	**0.9932**	0.9880	0.8684	0.8923	0.8401	0.9880	0.9876
#102	2	**0.9898**	0.9880	0.9383	0.9250	0.9250	0.9250	0.9250
	3	**0.9951**	0.9892	0.8768	0.8977	0.9097	0.9179	0.9179
	4	**0.9916**	0.9863	0.9256	0.9410	0.9050	0.9871	0.9871
	5	**0.9967**	0.9896	0.8446	0.9180	0.9181	0.9907	0.9895
#112	2	**0.9923**	0.9884	0.9356	0.9403	0.9404	0.9404	0.9404
	3	**0.9940**	0.9890	0.9147	0.9666	0.9769	0.9863	0.9890
	4	**0.9946**	0.9901	0.9751	0.9824	0.9825	0.9885	0.9896
	5	**0.9961**	0.9913	0.9735	0.9822	0.9830	0.9915	0.9914
